# Management of Calcium Channel Antagonist Overdose with Hyperinsulinemia-Euglycemia Therapy: Case Series and Review of the Literature

**DOI:** 10.1155/2012/927040

**Published:** 2012-02-14

**Authors:** Shiwan K. Shah, Sanjeev Kumar Goswami, Rajesh V. Babu, Gulshan Sharma, Alexander G. Duarte

**Affiliations:** ^1^Department of Internal Medicine and Pediatrics, University of Texas Medical Branch, 301 University Boulevard, Route 0354, Gaveston, TX 77555, USA; ^2^Department of Pulmonary, Allergy, and Critical Care, University of Texas Medical Branch, Gaveston, TX 77555, USA

## Abstract

Calcium channel antagonists (CCAs) are commonly involved in drug overdoses. Standard approaches to the management of CCA overdoses, including fluid resuscitation, gut decontamination, administration of calcium, glucagon, and atropine, as well as supportive care, are often ineffective. We report on two patients who improved after addition of hyperinsulinemia-euglycemia (HIE) therapy. We conclude with a literature review on hyperinsulinemia-euglycemia therapy with an exploration of the physiology behind its potential use.

## 1. Introduction

Calcium channel antagonists (CCAs) are widely prescribed for the treatment of cardiovascular diseases and have been demonstrated to be efficacious in the management of hypertension, cardiac arrhythmias, and angina. However, toxicity associated with overdose may produce serious, life-threatening complications, including bradycardia, hypotension, metabolic acidosis, and shock. In 2004, 10,513 cases of CCA toxicity were reported in the United States, resulting in 62 deaths [1]. Standard approach to the management of CCA overdose consists of intravenous fluid resuscitation, gut decontamination, administration of calcium, glucagon, and atropine, and supportive care. In severe cases, the development of bradycardia and hypotension may require placement of a temporary pacemaker and administration of vasopressors and inotropes. In many cases, however, the shock is refractory to inotropes and vasopressors, leading to cardiovascular collapse and death. Interestingly, recent case reports have described novel, successful management of CCA toxicity with euglycemic insulin therapy. We present our experience and review of the management of CCA toxicity with high-dose hyperinsulinemia-euglycemia (HIE therapy).

**Case 1**

A 40-year-old male with HIV, hepatitis C, and hypertension was admitted for hypotension, six hours after ingestion of approximately fifty extended release diltiazem pills in a suicide attempt (180 mg pills, 9 grams total). On arrival to the emergency department, vital signs were blood pressure of 70/40 mmHg; pulse of 87 min^−1^; respiratory rate of 35 min^−1^; temperature of 37°C and SpO_2_ 80% (FiO_2_ 0.50). The patient was intubated secondary to respiratory distress and was initially given 4 liters of intravenous normal saline followed by intravenous calcium and glucagon. Gut decontamination with activated charcoal administration via nasogastric tube was undertaken. Over the next several hours, additional fluids, glucagon, and calcium were given, with minimal improvement in the blood pressure or pulse: 75/42, pulse 82 min^−1^, respectively. Despite initiation of dopamine, norepinephrine, and epinephrine infusions, blood pressures did not improve, and he developed acute renal failure noted by a decrease in urine output and rise in creatinine from 1.1 to 2.7 mg/dL. Seventeen hours after the initial overdose, the patient was started on intravenous insulin, with no bolus and an initial rate of 10 units (0.11 units/kg). A 10% dextrose infusion was started and titrated to maintain an appropriate glucose concentration. The insulin infusion was increased over the next four hours to a max of 35 IU (0.4 units/kg) per hour. Within three hours after starting the insulin infusion, his blood pressure increased to 142/43 mmHg, and vasopressor requirements decreased. He was weaned off the epinephrine within 10 hours after starting the insulin infusion, weaned off the dopamine within 11 hours after starting the insulin infusion, and weaned off the norepinephrine within 21 hours after starting the insulin infusion. He was extubated 72 hours after initial presentation. The insulin drip was discontinued on the third hospital day following resolution of shock. No significant adverse effects were noted from the insulin therapy, and renal replacement therapy was not required.

**Case 2**

A 51-year-old African-American male with hypertension and bipolar disorder presented to our hospital after an intentional overdose of amlodipine, hydrochlorothiazide, bupropion, and quetiapine. Of note, he ingested approximately thirty amlodipine 10 mg tablets (total 300 mg). Initial physical exam revealed a lethargic, afebrile male with blood pressure 84/56 mmHg, pulse 92 min^−1^, respiratory rate 28 min^−1^, and SpO_2_ of 97% (FiO_2_ 0.21). Examination was notable for regular rate and rhythm and bilateral crackles on chest auscultation. Initial laboratory findings included a creatinine 3.2 mg/dL, glucose of 452 mg/dL, and serum lactic acid 6.9 mmol/L. Subsequently, an arterial blood gas obtained on a nonrebreather mask revealed a metabolic acidosis: pH 7.25, PaCO_2_ 33 mmHg, PaO_2_ 78 mmHg, bicarbonate 15 mEq/L. Chest radiographs showed bilateral interstitial infiltrates. He was given a total of 5 liters of normal saline, activated charcoal, and calcium chloride, but remained hypotensive with blood pressure 70/40 mmHg. He required intubation and mechanical ventilation. Despite administration of a norepinephrine infusion, and subsequently, phenylephrine infusion, he remained hypotensive. Continuous venovenous hemodiafiltration was initiated for renal failure. Thirty-six hours after presentation, intravenous insulin (no bolus, initial rate of 10 U/hr, 0.12 U/kg) and infusion of 10% dextrose (titrated to maintain an appropriate glucose concentration) were initiated. Insulin was titrated to 40 IU/hour (approximately 0.5 U/kg) and one day after insulin initiation, improvements in hemodynamics were noted. Over the following five days, the vasopressor and insulin infusion were gradually weaned off. He was extubated on hospital day 11 and transferred to the general medical floor where he continued to improve. After discharge, a serum amlodipine level obtained at time of admission was discovered to be 60 ng/mL (therapeutic levels 3–11 ng/mL). The only complication that developed as a result of therapy was hypophosphatemia, nadir of 0.9 mg/dL, which rapidly improved with phosphate replacement.

## 2. Discussion

Since being introduced almost fifty years ago, CCAs have become one of the most frequently prescribed class of medications. This significant usage has led to increasing reports of toxicity and in 2004, a national survey of poison control centers found 10,513 cases of CCA toxicity with 62 subsequent deaths [1].

### 2.1. Review of CCA Pharmacology

CCAs can be divided into two major categories: dihydropyridines and nondihydropyridines [2, 3]. Dihydropyridines (amlodipine, felodipine, nicardipine, and nifedipine) block L-type calcium channels, preferentially in the vascular smooth muscle, resulting in smooth muscle relaxation. These drugs have little myocardial depressant activity at therapeutic levels and in fact may increase cardiac output due to the reflex tachycardia. Nondihydropyridines (diltiazem and verapamil) block myocardial and smooth muscle L-type calcium channels, leading to myocardial depression and inhibition of electrical activity. However, two important points need to be noted. First, dihydropyridines are smooth muscle selective, not smooth muscle-specific, and in toxic concentrations may lead to myocardial depression and impaired cardiac conduction. Secondly, CCA, especially at high doses, can block sodium channels and can cause QRS prolongation, similar to tricyclic antidepressants.

In addition to actions on the heart and vascular smooth muscle, CCAs often have an effect on the pancreas. Calcium entry into the pancreatic beta cells via L-type calcium channels is essential for insulin release. Thus, CCA toxicity frequently results in hyperglycemia with relative hypoinsulinemia.

### 2.2. Clinical Findings of CCA Toxicity

Patients who overdose on CCAs often present with hypotension and bradycardia. The hypotension results from vasodilation and decreased cardiac output (due to the bradycardia and myocardial depression). The bradycardia is often secondary to sinus arrest, and patients often have an AV or ventricular escape rhythm. Due to the myocardial depression, patients may present with pulmonary edema. These patients often have an altered mental status, and they are often hyperglycemic due to insulin resistance, as mentioned earlier.

### 2.3. Management of CCA Toxicity

Management of CCA toxicity focuses on restoring cardiac function and systemic blood pressure (Table 2). Supportive care and gastrointestinal decontamination are the standard approaches. In addition, specific pharmacologic therapies available for CCA overdose include calcium, glucagon, adrenergic agents, and sodium bicarbonate. When pharmacological measures prove ineffective, cardiac pacing, intra-aortic balloon counterpulsation, and extracorporeal bypass may play important roles.

Initial therapy consists of securing the airway as many patients with CCA toxicity have an altered mental status. In addition, some of the therapies, such as glucagon, are associated with a significant risk of vomiting; thus, the importance of a secure airway cannot be overstated. In a hypotensive patient, an intravenous fluid bolus of 1-2 liters is warranted, especially in the absence of overt pulmonary edema. If hypotension persists, intravenous inotropes and vasopressors are warranted. Detoxification may include gastric lavage especially when a patient presents within 1-2 hours of ingestion. Other useful detoxification measures include the use of activated charcoal in patients within the first two hours of ingestion. However, especially with sustained release formulations, the use of activated charcoal up to 4 hours after ingestion has been documented to be effective. Lastly, whole-gut lavage with polyethylene glycol solution may be useful in selected cases of ingestion of sustained release tablets, although adverse outcomes have been reported with this treatment modality, especially in patients who are already hemodynamically unstable or who have ileus [4]. 

Intravenous calcium is a frequently used agent for calcium channel overdose. The goal is to competitively overcome the antagonism of the CCAs. However, not all patients respond to intravenous calcium administration, and the benefit may be temporary [5]. Calcium may be given either as calcium gluconate or calcium chloride. While calcium chloride contains three times more calcium for the same volume, calcium gluconate is less irritating to the veins and is preferred in most instances. Calcium salts can be given in bolus doses or administered as a continuous infusion [5]. A typical dosing would start with a 0.6 mL/kg bolus of calcium gluconate (0.2 mL/kg bolus of calcium chloride), followed by a continuous infusion of 0.6–1.5 mL/kg/hr of calcium gluconate (0.2–0.5 mL/kg/hr of calcium chloride), and the infusion rate titrated to hemodynamic response. Importantly, ionized calcium levels should be monitored, with the goal being two times the normal. While calcium salt administration is recommended for treatment of CCA toxicity, significant overdose with cardiovascular instability rarely responds to calcium as a single agent, and other measures are instituted.

Glucagon is another frequently recommended antidote for CCA overdose. Glucagon stimulates adenyl cyclase via G proteins, resulting in increased intracellular cyclic AMP which in turn leads to stimulation of muscle contraction. The clinical effect of glucagon resides in its positive inotropic and chronotropic effects as confirmed in multiple animal studies [6]. However, many of the results from animal studies have not been confirmed in human clinical trials [6]. The initial glucagon dose is 50–150 microgram/kg given as intravenous bolus or 3 to 10 mg in a 70 kg patient. The bolus may be repeated every 3–5 minutes to clinical effect, followed by infusions of the effective dose every hour. Main side effects of this therapy are nausea, vomiting, hyperglycemia, and ileus. The significant incidence of vomiting mandates ensuring a protected airway prior to glucagon administration.

 Sodium bicarbonate is another potentially useful therapy in treatment of CCA overdose. In an acidemic environment, CCA binding to the L-type calcium channel is increased. Thus, treatment of the acidemia may improve the hemodynamic status. In addition, CCAs, especially in high doses, may also inhibit fast sodium channels, leading to QRS prolongation, similar to tricyclic antidepressants [6]. If the QRS duration is longer than 120 milliseconds, a 1-2 mEq/kg bolus of sodium bicarbonate may be warranted.

Yet, in spite of the supportive care previously mentioned above, many patients often continue to experience clinical deterioration. In the past several years, hyperinsulinemia-euglycemia (HIE) therapy has gained wider acceptance as part of the treatment for CCA toxicity as described in multiple case reports and animal studies [7–21]. CCA toxicity often results in hyperglycemia from decreased insulin production due to the blockage of the L-type calcium channels in the pancreas. Another consequence of hypoinsulinemia is impairment of the myocardial energy supply. In most instances, the myocardium uses free fatty acids for energy. However, in a shock state, the myocardium switches to glucose use, dependent on insulin. With hypoinsulinemia and acquired insulin resistance, myocardial cells are unable to use glucose as an energy source, leading to decreased myocardial contractility and hypotension. HIE therapy may lead to reversal of cardiovascular collapse in CCA toxicity by improving myocardial utilization of carbohydrates as well as by clearing the cytosol of lactic acid and other glycolytic byproducts. In addition, insulin has direct positive inotropic activity that may contribute to its clinical effects [6, 22, 23]. 

Animal studies have indicated the role of insulin as an inotropic agent as well as the beneficial effects of insulin therapy in CCA poisoning. In a prospective randomized controlled trial, verapamil toxicity was induced in thirty mongrel dogs. The dogs were randomized to one of the following groups: control, calcium chloride, hyperinsulinemia-euglycemia, epinephrine, or glucagon. HIE treatment was reported to lead to larger increases in both myocardial glucose and lactate uptake thereby associated with improved cardiac performance. Importantly, all animals in the HIE group survived compared to 80% survival rate in the epinephrine group and a 60% survival rate in the glucagon group; a 50% survival in the calcium chloride groups and 0% in the control group [8].

However, no randomized controlled trials have yet been performed to evaluate the role of high-dose insulin therapy in human subjects with CCA overdose. Yet, hyperinsulinemia-euglycemia therapy appears to offer clinical benefit in management of acute myocardial infarction (AMI) and after coronary artery bypass grafting (CABG) surgery, although it goes by another name: glucose-insulin-potassium (GIK) therapy. A meta-analysis by Fath-Ordoubadi reported GIK therapy reduced inhospital mortality after acute myocardial infarction [24]. Another meta-analysis exploring the use of GIK in cardiac surgery, reported that GIK therapy may improve postoperative recovery of contractile function and reduce the incidence of atrial arrhythmias after CABG or heart valve replacement [25].

Most of the human data for HIE therapy in CCA toxicity is limited to published case reports [9–20, 26–29].  Table 1 summarizes the case reports and series in the literature that have used hyperinsulinemia therapy for CCA overdose in adults. These cases have involved the treatment of overdoses of diltiazem (9 cases), verapamil (10 cases), and amlodipine CCAs (9 cases). The dosing of the insulin bolus ranged from 0 to 1000 IU though only about half of the patients received a bolus, and the maintenance insulin infusion ranged from 0–2.64 IU/kg/hr. Current recommendations for the use of insulin therapy in CCA overdose consist of intravenous bolus administration of 1 IU/kg followed by an infusion of 0.5 IU/kg/hr. The duration of therapy ranged from 6 to 96 hours. In these reports and as well as our case series, hyperinsulinemia therapy resulted in hemodynamic improvement in the majority of treated patients, and 23 (82%) of the patients receiving therapy survived. Some authors have suggested that late onset of hyperinsulinemia-euglycemia is associated with lack of survival; however, in our patients, improvement was demonstrated up to 36 hours post presentation [6, 29]. 

Notably, hypoglycemia and hypokalemia are the main adverse effects of HIE therapy; therefore, serum glucose and electrolytes should be closely monitored. As suggested by Boyer, it is reasonable to administer 25 grams of glucose (1 ampule of D50) prior to initiation of HIE therapy if the blood glucose is less than 200 mg/dL, and similarly, to administer 40 mEq of potassium chloride intravenously if the potassium level is less than 2.5 meq/L. 

Although no definitive guidelines regarding HIE therapy in human CCA overdose have been published, there is enough empirical evidence to warrant strong consideration of this therapy for treatment of CCA overdose. More research is warranted to answer questions such as which patient populations would benefit most from this treatment, at what point in the treatment timeline should this therapy be instituted, and what are the optimal doses.

## 3. Conclusion

CCA poisoning is on the rise due to increased use for a number of cardiovascular indications. CCA overdose, whether intentional or accidental, can be lethal. HIE therapy has been shown to be beneficial in multiple animal studies as well as the majority of case series. HIE therapy should be considered early in the presentation of CCA toxicity in order to improve cardiac contractility and hemodynamics. Close monitoring of serum glucose and electrolytes is advised to prevent potential adverse effects.

## Figures and Tables

**Table 1:** Case reports of calcium channel antagonist overdose using hyperinsulinemia therapy with clinical outcomes.

CCA ingested	Dose range (mg)	Number of patients (%)	Insulin bolus (IU/kg)	Insulin infusion (IU/kg/hr)	Duration of treatment	Survival *N* (%)
Verapamil	2000–5800	10 (40)	0–1000 units (no IU/kg reported)	0-1	8–33 hours	9 (90)
Diltiazem	900–10080	9 (24)	0-1	0.2–1.5	6–8 hours	6 (67)
Amlodipine	30−1000	9 (36)	0-1	0–2.64	6–49 hours	8 (88)

**Table 2:** Typical treatment modalities.

(1) Decontamination/supportive therapy:
(a) activated charcoal: single dose of 50 g for adults;
(b) polyethylene glycol whole bowel irrigation: 2L/hr in adults until rectal effluent is clear;
(c) intravenous fluids;
(d) atropine: 1 mg IV (can be repeated up to 3 mg total).
(2) Antidotes:
(a) calcium salts:
(i) calcium chloride: 10–20 mL of a 10% solution administered over 10 min (can repeat dose if no effect);
(ii) calcium gluconate: 30–60 mL of a 10% solution (dose can be repeated if no effect);
(iii) continuous infusion with either salt: 0.5 meq of Ca/kg/hr;
(b) glucagon: 5 mg IV bolus, can be repeated twice at 10 min intervals.
(3) PDI (e.g., amrinone and milrinone).
(4) Adrenergic agents (e.g., norepinephrine and dopamine, etc.).
(5) HIE:
(a) regular insulin bolus of 0.1 U/kg IV and then continuous infusion of 0.2–0.5 U/kg/hr;
(b) dextrose 25 to 50 g bolus followed by a continuous infusion of 0.5 g glucose/kg/hr that can be titrated to appropriate blood glucose.
(6) Invasive therapy:
(a) transvenous pacing;
(b) intraaortic balloon pump;
(c) cardiopulmonary bypass;
(d) extracorporeal membrane oxygenation.
